# Memoir on Pericarditis, (Part the First) with Cases and Dissections

**Published:** 1830-07-01

**Authors:** 


### ST. GEORGE'S HOSPITAL.

### On Pericarditis.

The frequency of rheumatic pericarditis, its dangerous character, and the insidious manner in which it occasionally steals upon the patient, render an accurate knowledge of the disease an object of importance to the practical physician. Many excellent papers have been written on this subject, and yet it too frequently happens that the symptoms are not recognized by the practitioner, to the detriment of the patient and mortification or even disgrace of himself. We lately saw an instance in which two medical men, one in considerable practice, after carefully investigating the circumstances of the case, pronounced that the pericardium was not inflamed. On applying the cylinder, it was evident, and without auscultation it was more than probable, that the heart had been very severely inflamed, and enlargement of the organ with all the sequelæ of rheumatic pericarditis were fully established.

In the present report we propose to adopt the following arrangement. First we will give some fatal cases of rheumatic pericarditis; then some instances of pericarditis arising from the propagation of inflammation from the pleuræ, or from other causes; thirdly, cases of rheumatic pericarditis relieved by remedial treatment; fourthly, we will give the notes of two dissections, appearing to shew the perfect cure of the disease; and lastly, we will venture on a few remarks deduced from the facts we shall have laid before our readers, and from others which want of space may compel us to omit. We believe that by this method we shall exhibit in miniature a tolerable portrait of pericarditis.

### 1. Fatal Rheumatic Pericarditis.

Case 1.* Henry Hall, ætat. 33, a brush-maker, admitted July 15th, 1829, under Dr. Chambers.

Œdema of legs—dyspnœa—orthopnœa—much wheezing after exertion—startings from sleep—turbulent action of heart, which beats over a larger space than natural, with very distinct *bruit de soufflet*. Pulse 125, small, soft—skin cool—tongue slightly furred, moist—bowels open from medicine—urine free, natural.

Nearly two years ago had rheumatism for several months—has had palpitations and dyspnœa since last Christmas. In January last became affected with dropsy, for which he was in this hospital, and was benefited by the usual means. Has been getting worse for the last five weeks.

Blisters, blue pill with squill, diuretics, and one bleeding were the means employed, but the symptoms proceeded from bad to worse, and he died exhausted in the evening of the 27th.

*Sectio Cadaveris.* Body much emaciated—lower extremities rather œdematous.

---

* This case has been already detailed in a former number of the Journal; we shall therefore be very brief with it here.

*Thorax.* Pleuræ on left side universally united by old, elongated cellular adhesions—pleuræ on right side universally and almost inseparably adherent. Left lung crepitous, but not perfectly so, throughout, gorged with blood in its inferior parts, with serum in its superior—right lung more filled with blood and less crepitous than the left, and sufficiently dense in parts to sink in water. No tubercles in either lung.

Two layers of pericardium closely and completely bound together by old adhesions—no thickening of the membrane.

Heart very large, chiefly from hypertrophy and dilatation of left ventricle—cavities of other chambers dilated, but their parietes little altered in density. Valves and aorta sound.

*Abdomen.* Liver rather large, and of nutmeg colour in its interior—kidneys healthy.

*Cranium.* Not examined.

CASE 2. John Copas, æt. 24, a gardener, admitted Oct. 14th, 1829, under the care of Dr. Chambers.

Diffuse rheumatism, worse when warm and aggravated by sweating—slight œdema of legs—dyspnœa—orthopnœa—most extensive and strong action of heart, *agitating* nearly the whole chest—bruit de soufflet. Pulse 120, strong—skin cool, pallid—tongue clean—bowels open twice daily—urine free.

Had acute rheumatism eight years ago, and again four years ago, and heart has been affected since the first attack; has been able to work except at intervals, when he has suffered from attacks of dyspnœa with hæmoptysis—has generally lain on the left side in consequence of feeling uneasy on the right.

Blue pill with squill and digitalis, senna with supertartrate of potass, and a diuretic draught of nitric æther and juniper, &c. were prescribed, but the patient died suddenly on the morning after his admission.

*Sectio Cadaveris.* Surface generally leuco-phlegmatic and puffy—lower extremities œdematous.

*Thorax.* Pleuræ on both sides closely and universally united by old cellular adhesions. Lungs gorged with blood and serum in dependent parts, with air elsewhere, but not hepatized.

Layers of pericardium generally adherent, in some parts closely, in others more loosely—no fluid.

Heart of enormous size from hypertrophy and dilatation, nearly in equal proportions; hypertrophy of left ventricle very remarkable. Mitral valves slightly thickened and roughened but without bone; aperture not at all narrowed. Semilunar valves of aorta thickened; one of them shrivelled, and another so loosely connected at either end that it nearly hung back into the ventricle, and was incapable of presenting any barrier to the reflux of blood from the aorta.

Arch of aorta dilated without deposites—a few atheromatous patches in the descending thoracic at its commencement.

*Abdomen.* Liver rather large—kidneys natural.

*Cranium.* Clear serous effusion between arachnoid and pia mater—none in ventricles. Arteries of brain pretty healthy, carotids only being slightly opaque.

CASE 3. Mary Wood, æt. 18, unmarried, admitted Oct. 28th, 1829, under the care of Dr. Seymour.

Severe pain in chest, referred to lower part of left side, aggravated by deep inspiration, and preventing her from taking a full inspiration—cough without expectoration—dyspnœa—playing of alæ nasi—countenance pallid and rather anxious. Pulse 120—tongue white—bowels open.

Present symptoms commenced about a fortnight ago with severe pain below the left nipple; has been twice bled, blistered, and leeched, with some but not permanent relief. Two years ago had acute rheumatism for great part of Winter, and was leeched about the wrist, &c. Since that time she has suffered from shortness of breath on exertion, and been subject to palpitation, flatulence, and pain in the præcordia.

*V. S. ad* ℥xvj. *Hirud* xx. *pect. vesp. Postea fotus.*

*Hyd. sub.* gr. iij. *Op.* gr. ¼. *Conserv. cynosb. q. s. ut ft. pil. ter die sum.*

29th. P. 100, not strong—pain in left side of chest and hypochondrium—blood *not buffed or cupped;* (it was not got very freely.)

*H. Senn. stat. H. cetac. c. Tt. op.* ♏ xxv. *h. s. Interm. alia.*

31st. Complains of very severe pain in the side—tongue loaded—bowels not open.

*H. Senn. stat. Op.* gr. j. *Ext. hyos.* gr. ij. *ft. pil. h. s. s.*

On Nov. 1st, she referred her pain to the left false ribs and sternum, which parts were tender to the touch. Was ordered senna draught, warm bath in the evening, soap and opium pill with ext. col. c. every night, and a draught of ammoniated tincture of valerian in camphor mixture thrice daily.

On the 3d, when we examined her, she had dyspnœa—pain in left side of chest on inspiration—decubitus on that side—pulse quick and rather sharp—action of heart stronger than natural to the touch—arteries of neck beating strongly. By percussion, the dull sound in the region of the heart was extensive; by auscultation, the heart's action was heard more extensively than usual, especially on left side—the sound of the ventricle was prolonged, dull, and closely approaching to the bruit-de-soufflet—the impulsion was increased.

*Dilatation and hypertrophy of the heart, after pericarditis—probably adhesions of the pericardium.*

On the 5th a belladonna plaster was applied to the region of the heart, a draught of tinct. castor. with subcarbonate of ammonia ordered twice daily, and an anodyne pill prescribed at night. On the seventh these medicines were omitted and the patient was put upon calomel and opium, with occasional morning doses of senna. On the 10th the pulse was 120, sometimes intermitting, the cough troublesome, but unaccompanied with puriform expectoration. A blister was applied and ordered to be kept open, and on the 13th she felt relieved. On the 15th she complained of pain in the region of the heart increased by coughing—pulse sharp but not strong. Blister repeated—beef-tea and arrow-root. On the 17th she had no pain whatever, and the calomel was only exhibited at night; on the 19th the mouth was rather sore and the mineral was omitted altogether.

22d. Very ill—great anguish in countenance and urgency of respiration—action of heart tumultuous, heard over great space, and attended with decided *bruit de soufflet.* Opium at night and another blister. On the 23d the pain was relieved, but the aspect presented an extraordinary pallor; she lay on the right side on account of the blister, but otherwise preferred the left.

*Infus. digital.* ℥iij. *Sp. æth. nit.* ʒss. *Aq. pur.* ℥x. *Syrup. zing.* ʒj. *bis die. Rep. opium.*

A partial relief followed this alteration of treatment, but a sudden change for the worse took place in the night of the 26th, and on the next day she appeared to be sinking, and had all the symptoms of a person under the deleterious influence of digitalis. Æther mixture and brandy were employed, with the effect of arousing her from the state of collapse into which she had fallen, but the gleam was a brief one, the improvement fleeting, and on the 30th this unfortunate young woman expired. For a day or two before her death the left lower extremity was observed to be swollen and œdematous, but not painful, or at least not severely so.

*Sectio Cadaveris.* Body much emaciated—left lower extremity pitting on pressure.

*Thorax.* Pleuræ on both sides adherent anteriorly and laterally; posteriorly containing about a pint or rather less of serum. Bosom of either lung united to pericardium, and the latter attached by recent bands of lymph to the anterior wall of left side of chest. Right lung gorged with air above, with serum below; its lower lobe condensed and hepatized—left lung also filled with air and serum, and rather condensed inferiorly. No tubercles in either lung.

Two surfaces of pericardium inseparably and closely united; in some places a distinct layer of lymph interposed, in others the adhesions intimate, cellular, and organized.

Heart very large—cavities all dilated—parietes thickened, particularly those of the left ventricle. Lining membrane of left auricle remarkably opaque, and mitral valve so diminutive and shrunk that it *could not* have performed its office efficiently. Aorta and branches healthy. The heart generally from within presented a peculiar mottled appearance, not dependent on any morbid condition of its lining membrane.

*Abdomen*. Liver, kidneys, &c. healthy. Left common and external iliac, and femoral veins obstructed by a coagulum. This must have existed for some days, as it adhered very closely in parts to the internal tunic of the vein, and presented the appearance of softening or *pus* in its centre, occasionally observed in polypi of the heart. Coats of the vein not perceptibly thickened or inflamed—no mechanical or other apparent cause for the obstruction in its cavity.

*Cranium*. Not examined.

CASE 4.* John Green, æt. 43, admitted Jan. 6, 1830, under the care of Dr. Chambers.

"Weight in the chest" with dyspnœa and hoarseness—cough—thick and black sputa—emaciation—some palpitation. Pulse 120, sharp—skin cool, moist—tongue furred, yellowish white—thirst—bowels costive—urine scanty, and offensive.

Ailing three months. Was first attacked suddenly with dyspnœa; has been occasionally better, but is now worse than ever.

*V. S. ad* ℥xij.

*H. Sal.* c. *Oxymel Scill.* ʒss. 6*tis hor.*

*Cal.* gr. v. *h. n.* *Haust. Senn. cràs mane.* *Diæta parcissima.*

Vesp. *Empl. Canth. sterno.*

8th. Blood much buffed, highly inflamed—pulse 84, sharp—skin cool, moist—tongue furred—bowels costive—urine very free—expectoration of clear mucus tinged with blood.

*V. S. ad* ℥xij.

*Hyd. Sub.* gr. ij. *Op.* gr. ½, 6*tis hor.* *Haust. Senn. cràs.* *Omr. H. Sal.*

On the 9th he was seen by Mr. Hutchins, the house apothecary, and ordered leeches to the sternum, effervescing draughts, and a cathartic enema. In the evening a mustard poultice was put on the right side, and next day digitalis in infusion of roses.

11th. Vomited three pints of green bilious matter since yesterday evening—tenderness of right hypochondrium. Pulse 72, slightly hard—skin clammy—hiccup.

*Cal.* gr. iij.—*Op.* gr. ⅛, 3*tiis hor.*

*Cuc. Cr. ad* ℥x. *reg. hepatis. Posteà fotus.*

Next day he died. We examined this patient for a few moments with the stethoscope on the 8th. Arterial pulsation was *visible* at the root of the neck on either side, and above the sternum. The *bruit de scie* with some impulsion was distinct in the cardiac region. *These signs seemed to indicate active enlargement of the heart, and probably pericarditis.*

*Sectio Cadaveris.—Thorax.* Right side apparently more prominent than the left; old adhesions of pleuræ in the latter, and about three ounces of serum—about eight ounces in the former. Lungs œdematous and emphysematous—lower parts of both carnified.

Pericardium containing organized lymph, partly disposed in the form of bands, partly as a flocculent deposite.

Cavities of heart, more particularly of right ventricle considerably dilated; no hypertrophy of latter. Much hypertrophy of left ventricle, its parietes being three fourths of an inch in thickness at base, half an inch at apex. Valves natural — slight atheromatous deposites round the coronary arteries.

*Abdomen*. No marks of hepatic inflammation—kidneys, &c. natural.

*Cranium*. Not examined.

---

* The history of this case was imperfectly obtained, and consequently the presence or previous existence of rheumatism was not ascertained. From the extreme rarity, however, of idiopathic pericarditis, we have thought it safer to introduce it in its present place, than to offer it, without better proof, as an instance of so unfrequent a form of the disease.

### II. Pericarditis succeeding Disease or Inflammation in contiguous Parts.

Case 5. *Pericarditis — aneurismal pouch at the origin of the aorta.*

Henry Cook, æt. 29, a stone-sawyer, admitted Dec. 9, 1829, under the care of Dr. Chambers.

Pain across chest, especially in left side, increased by full inspiration which excites cough—dyspnœa on the least exertion—palpitation—throbbing of the head—decubitus on left side difficult. Pulse 120, full and sharp—skin cool—tongue white—some thirst—anorexia bowels costive—urine high-coloured.

Has been disabled by illness from working for the last six weeks—was at first seized with pain in the epigastrium, which extended to the left side. Says he has not been feverish.

*V. S. ad deliq. et rep. post hor.* 8 *nisi prius cessårit dolor.*

*H. Salin.* c. *Liq. Ant. T.* ʒj. 6*tis. hor.*

*H. Sennæ cràs—D. Parcissima.*

10th. Has been bled to ℥xxx with some relief at the time but more this morning—still some pain in left side. Pulse 110, softer — tongue furred — blood buffed.

*C. C. lat. Sinist. ad* ℥xij.

*Hyd. Sub.* gr. v. *o. n. P.* c. *H. Salin.*

11th. Relieved by cupping—deep inspiration still excites pain and slight cough—heart tranquil—pulse 96, soft —bowels open—urine scanty and high-coloured. Occasional chills succeeded by heat.

*Empl. Canth. amplum lat. dol.*

*Adde Haust. Tr. Op.* ♏vi. *H. Senn. cràs.*

14th. More tightness across left side and dyspnœa—inspiration causes dry cough — starts at night — pulse 120, sharp—slight thrill in action of heart.

*V. S. ad* ℥xiv. *et peract. V. S. capt. Op.* gr. ii.

*Rep. H. Senn. cras mane et H. Sal.* &c.

15th. Sinking. Stimuli were given but with no avail, for he rallied not and died in the course of the morning.*

*Sectio Cadaveris.* Body not much emaciated.

*Thorax.* Pleuræ on right side extensively united by old cellular adhesions; a little fluid in the posterior part of the cavity—pleuræ on left side not so extensively adherent; slight flakes of recent lymph on pleura pulmonalis, and more fluid than on opposite side. Lungs gorged with serum in upper lobes, carnified in lower.

Pericardium containing about 4 ozs. of straw-coloured clear fluid, with some bands of recent lymph extending across its cavity, particularly at the root of the aorta, where the two surfaces of the membrane adhered pretty firmly.

Some dilatation and more hypertrophy of the left ventricle of the heart—right ventricle rather thicker than natural. Valves sound.

Immediately above the semilunar valves the aorta presenting an aneurismal sac about the size of a duck's egg, covered in great measure by the united layers of the pericardium, but projecting in part within that cavity. The communication between the sac and the aorta was about an inch in diameter, and the margins of the aperture so abrupt and defined that the sac was evidently that of a false aneurism. The wall of the sac on one side was formed by the right side of the pulmonary artery, and this was so thin that it had either given way spontaneously or been accidentally injured in the examination, for a small rent was found in it by which a communication was established between the sac and the pulmonary artery. The semilunar valve of the pulmonary artery below the fissure was firmly united to the side of the vessel.

Dilatation of ascending arch of aorta —some atheromatous deposites in the coats.

*Abdomen.* Nothing particular.

*Cranium.* Not examined.

Case 6. *Pericarditis—Aneurism of the Aorta at its Origin.* Thomas Hill, æt. 42, a paper-hanger, admitted last Autumn, under the care of Dr. Wilson.

---

* We did not see this patient during life, nor, as far as we know, was the stethoscope applied.

On right side of inferior half of sternum a globular-shaped tumour, slightly prominent, not distinctly circumscribed, pulsating synchronously with the arterial pulse. Pulsation stronger and more superficial at two particular spots —tenderness of the integuments on pressure—tenderness over sternum and contiguous ribs. Pain shooting down right arm—occasional swelling of the same—puffiness of face—disposition to œdema of legs. Dyspnœa—some orthopnœa—not much cough—palpitations—flatulence. Pulse sharp, vibratory—face rather florid and injected—urine scanty.

Attributes his complaint to over-exertion at his business a twelvemonth ago, shortly after which he became affected with constant darting pains in right side of thorax, increased upon exertion and attended with dyspnœa. About nine months ago first noticed the tumour, and about that time the swelling of the legs and palpitations first made their appearance.

*Auscultation.* Strong bruit de soufflet with much impulsion in tumour; former heard over a large space—stroke above right clavicle clearer than in tumour. Heart's action powerful, and accompanied with bruit de soufflet, not so whizzing as in tumour—auricular sound rough as well as ventricular.

*Active enlargement of heart, especially ventricles. Aneurism of aorta very near its rise from the left ventricle, part of sac probably receiving a covering from the pericardium—depositions in coats of aorta—some dilatation of aorta beyond aneurismal sac.*

The patient went on tolerably well from the time of his admission till about the middle of November. He was then attacked with cynanche tonsillaris, the right knee-joint swelled, erysipelas of the face, with much prostration, supervened, and on the 21st the patient died.

*Sectio Cadaveris.* Face puffy—lower extremities slightly œdematous.

*Thorax.* Pleuræ on right side united by old adhesions—pleuræ in upper two-thirds of left side united in the same manner, but containing below a few ounces of dark, turbid, sero-purulent fluid. Bosom of right lung attached to the side of the aneurismal tumour, which projected into the right side of the chest; lung in this situation carnified. Lower lobe of left lung carnified also.

Surfaces of pericardium united closely and universally by old cellular adhesions. Heart actively enlarged, especially the left ventricle, but not to an extreme degree.

Whole arch of aorta much dilated—coats puckered and uneven from atheromatous deposites, without any bone—internal coat generally sound. From the right side of the aorta, at its root, arose a pouch sufficiently large to contain a russet apple. It passed under the right border of the sternum, encroached on the right pleural cavity, and its superficial paries was in intimate connexion with the cartilages of the 2d, 3d, and 4th ribs. None of the cartilages destroyed. Sac nearly filled with a polypoid concretion, not laminated, and only partially adhering to its sides. Inner surface, both of the sac and dilated aorta, fissured and abraded in several places.

*Cranium and Abdomen.* Nothing particular.

*Right Knee-joint.* An ounce or more of pus in the cavity of this joint, without abrasion or ulceration of the cartilages.

CASE 7. *Pericarditis—Fungus Hæmatodes of the left Lung, and Pleurisy.**

Benjamin Long, æt. 27, admitted, Dec. 16, 1830, under the care of Dr. Hewett.

Cough—cutting pain in left side—decubitus on that side—inspiration imperfect and wheezing—expectoration of glairy, scanty mucus—emaciation—disposition to hectic.

First attacked, 6 weeks ago, with cough, dyspnœa and pain, frequent chills, succeeded by fever and thirst. For first fortnight spat some scarlet

---

* We shall notice this and the two succeeding cases very briefly, as they will, with one exception, form the subject of a future report for a different purpose.

blood. After three weeks' illness convalesced, but a fortnight ago had a relapse, and has since suffered from present symptoms. Was previously a healthy man.

On a subsequent and more careful examination of the chest, the left side was found to be contracted, universally dull on percussion, and devoid of respiratory murmur. The heart, too, was heard and seen acting more extensively than natural, but without increased impulsion or bruit de soufflet. On the 13th of March, examination of the heart by the stethoscope discovered more impulsion and slight bruit de soufflet. On the 24th, the left side of the chest, which had previously measured an inch less than the right, was found to have regained its natural dimensions. On the 30th, the patient was seized with a rigor and vomiting of glairy mucus, pain in the stomach and wildness of manner. Next day he was attacked with severe pain in the left side of the chest, the intercostal spaces were raised to the level of the ribs, ægophony was established, and this side now measured three quarters of an inch *more* than the right. On the 3d the patient died.

*Sectio Cadaveris.* Body emaciated.

*Thorax.* Some old cellular adhesions between pleuræ on right side—no fluid. Some crude tubercles and one very small vomica in right lung. Partial adhesions of pleuræ on left side, with a pint or more bloody serum and a few flakes of lymph. This effusion, from its comparative clearness, was evidently of recent date. Left lung almost universally infiltrated with deposites of fungus hæmatodes, not encysted. The great bronchus on this side was pressed on by the malignant growth, its channel nearly obstructed, and its parietes assimilated to the disease. No part of the lung respirable in the least degree. One of the malignant deposites had pressed on the pericardium opposite the left auricle, and although the membrane was not ulcerated or destroyed, the morbid growth encroached on the cavity.

Considerable quantity of serum, with flakes of lymph, in the cavity of the pericardium, the opposite surfaces of which were more opaque than natural. The membrane was corrugated, and in some places slightly reticulated.

Heart itself not enlarged or otherwise diseased—great vessels healthy.

Case 8. *Slight Pericarditis—Pneumo-thorax on right Side.*

James Smith, æt. 21, a footman, admitted March 8th, 1830, under the care of Dr. Hewett.

Pain under lower half of sternum, which is not raised by inspiration—dyspnœa—some cough—no expectoration. Decubitus on *left* side, but is obliged to change his position frequently in consequence of cough. Pulse 140, very small—aspect pallid and strumous.

Ill six days, but had a bad cold previously. Was first seized suddenly with severe pain in the lower part of right side of chest, for which he has been actively treated. Has been subject to winter cough.

On the 10th he was examined by the stethoscope, and the presence of pneumo-thorax with bronchial communication discovered without difficulty. The dyspnœa, &c. increased, the right side became larger than the left, the hectic was severe, and to render the case even more hopeless than before, the absorbents in the left arm became inflamed in consequence of the puncture made in bleeding before his admission into the hospital. The contractions of the heart were extremely rapid, but no bruit de soufflet existed, or at least we discovered none. On the 4th of April the patient died.

*Sectio Cadaveris.* Pneumo-thorax with about a quart of green sero-purulent fluid in the right side of the chest. Pleuræ coated with "concrete pus," and presenting in one or two parts some long bands of adhesion between them. At the inferior part of the middle lobe of the lung, very near the spine, a fistulous opening leading into a vomica about the size of a horse-bean, and communicating through it with a large bronchial tube. Lung itself generally carnified. Pleuræ on left side free from inflammation, but united by some old adhesions. Lung containing some tubercles, and one or two very small vomicæ.

Heart pushed over towards the left side. About an ounce and half of greenish serum in the pericardium, with some flocculi of loose recent lymph. Heart not at all enlarged, or altered in structure.

*Abdomen.* Some inflammation of the peritoneum covering the diaphragm and liver.

*Cranium.* Not examined.

Case 9. *Chronic Pericarditis—Pneumo-thorax on Right Side.** George Canning, æt. 23, a gardener, admitted Nov. 11th, 1829, under the care of Dr. Chambers.

This patient had pain in the right side of the chest, dyspnœa, cough, hectic. He had been subject to cough for some time, and had suffered from the more severe symptoms for eleven days. They had been combated by active depletory measures. On applying the stethoscope the signs of pneumo-thorax in the right side were apparent. He remained in the house till the 19th of January when he died. During the interval between his admission and decease the cough and hectic had continued, the expectoration was never more than *suspicious* and always scanty, he was successively attacked with pain in both sides of the chest and in the præcordia, his dyspnœa was occasionally severe, and latterly he had some orthopnœa likewise. He never complained of palpitations, nor was the stethoscope applied after the day of his admission.

*Sectio Cadaveris.* Pneumo-thorax, liquid effusion, and fistulous communication with bronchi in right side of chest. Thick layer of lymph and concrete pus on pleuræ. Lung compressed and carnified. Old adhesions of pleuræ on left side—several groups of miliary tubercles in left lung.

Heart pushed considerably to the left. A quantity of bloody serum in the pericardium, and reticulated lymph, not very recent, deposited on both its surfaces. Heart little, if at all enlarged, and rather flaccid. Valves and great vessels healthy.

* This case having already been detailed in a former number, we shall merely glance at it.

We had hoped and intended to have completed the article in this Fasciculus, but we find it is impossible. We shall therefore stop for the present, and commence the succeeding portion of the report, with cases of "rheumatic pericarditis successfully treated." It is not for deficiency of stores that we are now brought to anchor, but for want of sea-room. H. J.

